# Low-Complexity Parallel Decoding for Non-Autoregressive Learned Image Compression

**DOI:** 10.3390/e28101134

**Published:** 2026-10-15

**Authors:** Linghao Zhang, Qing Zhang, Bo Pang, Siyu Xiang, Wenjuan Wu

**Affiliations:** 1 State Grid Sichuan Electric Power Research Institute, Chengdu 610043, China; 2 Sichuan Provincial Key Laboratory of Security and Operation for New-Type Power Systems, Chengdu 610043, China; 3 State Grid Sichuan Electric Power Company, Chengdu 610041, China; 4 Aostar Information Technologies Co., Ltd., Chengdu 610200, China

**Keywords:** learned image compression, non-autoregressive entropy model, parallel decoding, lightweight neural network, activation sparsity

## Abstract

Autoregressive probability models improve the rate–distortion performance of learned image compression, but their spatial conditioning introduces serial neural inference and high decoding latency. This paper presents a latency-oriented learned image compression framework that jointly addresses sequential entropy estimation, decoder computation, and dense intermediate activation responses. First, a dependency-decoupled entropy model estimates all latent distribution parameters in parallel from hyperprior statistics, eliminating the neural autoregressive loop. Second, a lightweight synthesis transform uses compact convolutional mappings, reduced channel widths, and a short progressive reconstruction path. Third, a differentiable activation-magnitude regularizer penalizes the normalized $L_{1}$ magnitude of intermediate decoder activations and is introduced gradually during rate–distortion training to encourage sparse feature responses. The activation regularizer is treated as a representation-level training constraint rather than as a direct estimator of dense-convolution arithmetic. Experiments on Kodak and BSD100 show that the proposed model consistently improves on the hyperprior baseline over the evaluated bitrate range. On Kodak, it achieves BD-rate reductions of 7.9% and 9.7% relative to hyperprior in terms of PSNR and MS-SSIM, respectively. At approximately 0.30 bpp, the proposed model achieves 32.94 dB PSNR and 0.9745 MS-SSIM with 8.10 M parameters, 165.3 GMACs, and a decoding time of 35.6±1.0 ms. Compared with the serial autoregressive joint baseline at a comparable operating point, this corresponds to a 95.0% reduction in decoding latency.

## 1. Introduction

The rapid growth of networked multimedia, computer vision, and edge applications has increased the demand for efficient image compression to reduce storage and transmission costs. Traditional standards such as JPEG and BPG rely on hand-crafted transforms, quantization, and entropy coding [1,2]. Although these codecs are mature and computationally predictable, their coding pipelines are largely based on manually designed transforms and entropy-coding tools.

End-to-end learned image compression provides a data-driven alternative in which an analysis transform maps an image to a latent representation, quantization and entropy coding produce a compact bitstream, and a synthesis transform reconstructs the image. Hyperprior-based models substantially improve latent probability estimation [3], while autoregressive and hierarchical priors further exploit latent dependencies at the cost of sequential decoding [4]. Subsequent studies have explored more efficient spatial and space–channel context structures [5,6], together with alternative entropy modeling strategies based on wavelet-domain representations and dictionary-based dependencies [7,8].

Recent work has increasingly addressed the decoding inefficiency of context-based learned compression. The Checkerboard Context Model reorganizes spatial context prediction into parallelizable passes, avoiding the strict raster-scan order of conventional autoregressive decoding [5]. ELIC further considers compression efficiency and runtime through unevenly grouped space–channel contextual coding and an efficiency-oriented transform architecture [6]. More recently, Fu et al. employ dual-resolution latent representations together with channel-wise conditional entropy coding to reduce dependence on serial spatial context modeling [9]. These studies establish parallel entropy modeling and efficient transform design as important directions for reducing LIC decoding complexity. Motivated by these observations, we consider three sources of decoding inefficiency together: sequential entropy estimation, decoder computation, and dense intermediate activation responses.

Improved probability estimation often comes at the cost of increased decoding latency. Spatial autoregressive models predict one latent symbol from previously decoded symbols, creating sequential dependencies that limit the utilization of parallel hardware [4,5]. Deep synthesis networks and complex attention blocks can add further parameters and arithmetic operations. These limitations are particularly important in latency-sensitive and resource-constrained applications. We therefore evaluate reconstruction quality, model complexity, activation sparsity, and measured decoding latency as separate quantities.

These inefficiencies arise at different stages of the decoding pipeline. Reducing the sequential dependency of entropy estimation does not necessarily reduce the arithmetic cost of image reconstruction, whereas simplifying the synthesis transform alone does not eliminate serial context inference. In addition, architectural complexity measures do not characterize the magnitude distribution or sparsity of intermediate activations. These factors therefore need to be evaluated separately within the same decoding system.

The main contributions of this work are as follows:

1. At the dependency level, we remove position-wise neural conditioning on previously decoded latents and use a hyperprior-conditioned factorization to generate all latent distribution parameters in a single neural forward pass. This eliminates repeated position-dependent context-network inference and allows entropy parameters to be computed in parallel.
2. At the architecture level, the dependency-decoupled entropy path is paired with a compact synthesis transform using reduced channel widths and a shortened progressive reconstruction path. This design reduces the parameter count and nominal arithmetic cost of image reconstruction.
3. At the optimization level, the conventional rate–distortion objective is extended with a normalized activation-magnitude regularizer that penalizes intermediate activation magnitudes and encourages sparser feature responses. This regularizer acts only during training and does not change the inference topology or introduce an additional inference branch.

Together, these components target different sources of decoding cost while allowing compression quality, model complexity, activation sparsity, and measured latency to be evaluated independently.

## 2. Methods

### 2.1. Overall Architecture

Let x denote an input image. The analysis transform $g_{a}$ produces a latent tensor $y=g_{a}\left(x\right)$, and the hyper-analysis transform $h_{a}$ extracts a hyperlatent tensor $z=h_{a}\left(y\right)$. During training, quantization is approximated by additive uniform noise, $\tilde{y}=y+u_{y}$ and $\tilde{z}=z+u_{z}$, where $u_{y},u_{z}∼U\left(-\frac{1}{2},\frac{1}{2}\right)$. During evaluation and entropy coding, hard quantization is used. The hyperlatents are quantized as $\hat{z}=\mathrm{round}\left(z\right)$, whereas the primary latents are quantized relative to the predicted conditional mean as $\hat{y}=\mathrm{round}\left(y-\mu \right)+\mu$. The two entropy-coded streams form the compressed representation. During decoding, the hyperlatent stream is decoded first, the hyper-synthesis network predicts the probability parameters of $\hat{y}$, and the lightweight synthesis transform reconstructs $\hat{x}$ from $\hat{y}$. Figure 1 summarizes the architecture.

The decoding pipeline contains three components with different roles. The entropy model determines the dependency structure of probability estimation, the synthesis transform determines the parameter count and nominal computation required for reconstruction, and the activation regularizer acts on intermediate decoder activations during training.

### 2.2. Dependency-Decoupled Non-Autoregressive Entropy Model

In a conventional joint entropy model, the probability of a latent symbol depends on previously decoded symbols:

(1) $$p\left(\hat{y}|\hat{z}\right)=\prod_{i}p\left({\hat{y}}_{i}|{\hat{y}}_{<i},\hat{z}\right).$$

Although this factorization can improve probability estimation, it requires O(N) sequential neural predictions for *N* latent positions. Within the proposed framework, the dependency-level modification removes the spatial context input and conditions the latent probability model only on the decoded hyperprior. This dependency-decoupled component is referred to as non-autoregressive entropy modeling (NAE) in the subsequent ablation and sensitivity analyses. The hyper-synthesis transform generates the mean and scale fields in a single pass:

(2) $$\left(\mu ,\sigma \right)=h_{s}\left(\hat{z};\theta_{h}\right),$$

where $\theta_{h}$ denotes the parameters of the hyper-synthesis network. Each quantized latent is modeled using a discretized Gaussian likelihood obtained by convolving the Gaussian density with a unit-width uniform density:

(3) $$p_{{\hat{y}}_{i}}\left({\hat{y}}_{i}|\hat{z}\right)=\left[N\left(\mu_{i},\sigma_{i}^{2}\right)*U\;\left(-\frac{1}{2},\frac{1}{2}\right)\right]\left({\hat{y}}_{i}\right).$$

Conditioned on $\hat{z}$, the latent positions are therefore treated as independent:

(4) $$p\left(\hat{y}|\hat{z}\right)=\prod_{i}p\left({\hat{y}}_{i}|\hat{z}\right).$$

All parameter fields can be evaluated concurrently on a GPU. The estimated latent rate is

(5) $$R_{\hat{y}}=E\;\left[-\log_{2}p\left(\hat{y}|\hat{z}\right)\right].$$

The term *parallel decoding* refers here to parallel neural parameter generation and parallel forward inference. It does not imply that an arithmetic-coded bitstream can be read in arbitrary order. Figure 2 contrasts the eliminated neural autoregressive loop with the proposed one-pass computation.

### 2.3. Lightweight Decoder Architecture

The synthesis transform maps the decoded latent tensor to the reconstructed image:

(6) $$\hat{x}=g_{s}\left(\hat{y};\theta_{d}\right),$$

where $\theta_{d}$ denotes the decoder parameters.

The synthesis transform is simplified to reduce the parameter count and nominal computation required for image reconstruction. The decoder uses a shallow sequence of compact convolutions and progressive upsampling blocks (Figure 3). This short reconstruction path reduces the number of feature transformations while keeping each stage fully convolutional.

The layer configuration of the synthesis transform is summarized in Table 1.

For each pixel shuffle stage with scale factor 2, the convolution preceding pixel shuffle produces $4C_{l}$ channels, which are rearranged by pixel shuffle into $C_{l}$ output feature channels at twice the spatial resolution. Thus, the channel values reported in Table 1 denote the feature channels after pixel shuffle.

For a two-dimensional convolutional layer *l* with kernel size $k_{l}\times k_{l}$, $C_{l}^{\mathrm{in}}$ input channels, and $C_{l}^{\mathrm{out}}$ output channels, the trainable parameter count, including the bias term, is

(7) $$P_{l}=k_{l}^{2}C_{l}^{\mathrm{in}}C_{l}^{\mathrm{out}}+C_{l}^{\mathrm{out}}.$$

The synthesis transform parameter count is therefore

(8) $$P_{\mathrm{dec}}=\sum_{l=1}^{L_{\mathrm{dec}}}P_{l}.$$

For a pixel shuffle block with scale factor 2, $C_{l}^{\mathrm{out}}$ in the parameter and MAC calculations denotes the number of convolution output channels before pixel shuffle, i.e., $4C_{l}$ when the post-shuffle feature width is $C_{l}$. Reducing network depth and channel width therefore decreases both the synthesis transform parameter count and nominal convolutional GMACs. This architectural change is independent of the entropy parameterization mechanism.

### 2.4. Rate–Distortion Objective with Activation Regularization

Conventional learned compression minimizes a rate–distortion objective,

(9) $$L_{\mathrm{RD}}=R+\lambda D,$$

where *R* is the expected bit rate, *D* is the reconstruction distortion, and λ controls their trade-off. The total rate includes the primary and hyperlatent streams:

(10) $$R=E\;\left[-\log_{2}p\left(\hat{y}|\hat{z}\right)\right]+E\;\left[-\log_{2}p\left(\hat{z}\right)\right].$$

Mean-squared error is used for distortion and is computed over all color channels and spatial locations:

(11) $$D=\frac{1}{CHW}\sum_{c=1}^{C}\sum_{m=1}^{H}\sum_{n=1}^{W}{\left(x_{c,m,n}-{\hat{x}}_{c,m,n}\right)}^{2}.$$

The entropy model modification changes the decoding dependency, whereas the lightweight synthesis transform changes the reconstruction architecture. In contrast, activation regularization acts only during training and does not alter the inference topology. We therefore define an activation-magnitude regularizer

(12) $$C_{a}=\sum_{l=1}^{L_{c}}\frac{1}{H_{l}W_{l}C_{l}}{∥a_{l}∥}_{1},$$

where $a_{l}\in R^{H_{l}\times W_{l}\times C_{l}}$ denotes the feature tensor sampled at the output of constrained decoder block *l* after its nonlinear activation and before the subsequent spatial upsampling operation; $H_{l}$, $W_{l}$, and $C_{l}$ denote its height, width, and channel number, respectively. The complete training objective is

(13) $$L=R+\lambda D+\alpha C_{a}.$$

The activation term $C_{a}$ is a training regularizer rather than a direct estimator of executed arithmetic operations. In a conventional dense-convolution implementation, reducing the magnitude of an activation does not change the nominal number of multiply–accumulate operations because the same dense kernels are executed regardless of whether individual responses are close to zero. Consequently, activation sparsity, nominal GMACs, and measured inference latency are treated as separate quantities throughout the evaluation. Actual acceleration attributable to sparsity would require an execution backend capable of exploiting sparse or zero-valued responses and is not assumed for the standard dense GPU implementation used in this study.

To avoid imposing activation regularization during the early stage of rate–distortion training, the coefficient α follows a staged schedule:

(14) $$\alpha \left(t\right)=\left\{\begin{array}{cc} 0, & t<T_{0},\\ \alpha_{\max}\frac{t-T_{0}}{T_{1}-T_{0}}, & T_{0}\leq t<T_{1},\\ \alpha_{\max}, & t\geq T_{1}, \end{array}\right.$$

where *t* denotes the training step. In the training configuration used for the reported experiments, activation regularization is introduced at $T_{0}=2.0\times 10^{5}$ steps, linearly increased until $T_{1}=4.0\times 10^{5}$ steps, and then held at $\alpha_{\max}=1.0\times 10^{-4}$ for the remainder of training. These values are fixed before evaluation on Kodak and BSD100 and remain unchanged during test set evaluation.

### 2.5. Experimental Setup

#### 2.5.1. Hardware, Software, and Training

All models are trained and evaluated on the same hardware and software platform. Table 2 summarizes the experimental configuration.

The models are optimized using Adam with $\beta_{1}=0.9$, $\beta_{2}=0.999$, and zero weight decay. The initial learning rate is $10^{-4}$ and is reduced to $10^{-5}$ after $8.0\times 10^{5}$ optimization steps; training continues for a total of $1.0\times 10^{6}$ steps. The batch size is 16. Training images are randomly cropped into 256×256 patches and augmented using random horizontal and vertical flips with probability 0.5.

A separate model is trained for each rate–distortion coefficient λ to obtain multiple operating points. Five coefficients are used and are denoted by

(15) Λ={0.0035,0.0067,0.0130,0.0250,0.0483},

All rate points use the same training dataset, optimizer, learning rate schedule, augmentation strategy, and stopping criterion.

For models using activation regularization, the coefficient α follows Equation (14) with $T_{0}=2.0\times 10^{5}$, $T_{1}=4.0\times 10^{5}$, and $\alpha_{\max}=1.0\times 10^{-4}$. These values are fixed before test set evaluation and are not selected according to Kodak or BSD100 performance.

The training configuration uses the Open Images training partition [11]. A fixed subset of 300,000 images is used for optimization, and a disjoint set of 5000 images is reserved for validation. Each of the five rate points is trained for $1.0\times 10^{6}$ optimization steps using the same data split, augmentation pipeline, optimizer, and stopping criterion. The selected checkpoint is the model with the lowest validation rate–distortion loss after the learning rate decay.

#### 2.5.2. Test Datasets

Kodak is used as the primary evaluation set [12], and BSD100 provides an additional evaluation set [13]. Kodak contains high-quality natural scenes with smooth areas, edges, and moderate textures and is widely used in learned compression. BSD100 contains 100 natural images with diverse textures and boundary structures, providing an additional test of reconstruction performance across different image content.

Kodak and BSD100 are evaluated independently, and results from the two datasets are not pooled into a single average. Rate–distortion curves are constructed separately for each dataset over the common bitrate range covered by the compared models. Rate–distortion performance is evaluated over all available operating points, whereas the operating point near 0.30 bpp is used for detailed complexity and latency comparisons.

#### 2.5.3. Compared Methods

JPEG [1] and BPG [2] are introduced as conventional coding references in the background discussion. The quantitative cross-method evaluation in this study focuses on learned image compression methods. The scale-hyperprior model of Ballé et al. [3] and the joint autoregressive–hierarchical model of Minnen et al. [4] are retained as canonical learned-compression baselines.

Representative recent methods included in the comparison are the Checkerboard Context Model (CCM) [5], ELIC [6], the dual-branch conditional coding model of Fu et al. [9], and the Dictionary-based Entropy Model [8]. These methods cover parallel spatial-context modeling, efficiency-oriented transform design, reduced serial context dependence, and recent entropy modeling strategies.

Rate–distortion performance is evaluated over multiple operating points on Kodak and BSD100 separately. The hyperprior and joint methods are instantiated through the CompressAI 1.2.2 implementations used in the present environment. The remaining recent learned baselines are executed locally using the corresponding implementations and checkpoints adopted for this study; their architectural settings are matched to the cited publications. All learned models are decoded under the same hardware, software, input resolution, batch size, and timing protocol. Consequently, every latency value reported in this paper is remeasured locally, and no runtime value is copied directly from the original publications.

#### 2.5.4. Evaluation Metrics

For learned codecs, bitrate is computed from the actual entropy-coded payloads of both the primary latent stream and the hyperlatent stream. Let $S_{y}$ and $S_{z}$ denote their encoded byte lengths. The reported payload size is

(16) $$S_{c}=S_{y}+S_{z},$$

and bits per pixel are computed as

(17) $$\mathrm{bpp}=\frac{8S_{c}}{HW}.$$

External container headers, file system metadata, and source image headers are excluded from $S_{c}$ for all learned methods. The same payload-only convention is used for every learned codec.

For a *C*-channel image, the pixel-domain mean-squared error and PSNR are

(18) $$\begin{array}{cc} \mathrm{MSE} & =\frac{1}{CHW}\sum_{c=1}^{C}\sum_{m=1}^{H}\sum_{n=1}^{W}{\left(x_{c,m,n}-{\hat{x}}_{c,m,n}\right)}^{2}, \end{array}$$

(19) $$\begin{array}{cc} \mathrm{PSNR} & =10\log_{10}\left(\frac{{\mathrm{MAX}}_{I}^{2}}{\mathrm{MSE}}\right), \end{array}$$

where ${\mathrm{MAX}}_{I}$ is 255 for 8-bit images or 1 for normalized images. Multi-scale structural similarity is

(20) $$\mathrm{MS}\text{-}\mathrm{SSIM}\left(x,\hat{x}\right)={\left[l_{M}\left(x,\hat{x}\right)\right]}^{\alpha_{M}}\prod_{j=1}^{M}{\left[c_{j}\left(x,\hat{x}\right)\right]}^{\beta_{j}}{\left[s_{j}\left(x,\hat{x}\right)\right]}^{\gamma_{j}},$$

following Wang et al. [14].

All PSNR and MS-SSIM values are computed on reconstructed RGB images in the normalized [0,1] range without border cropping. The same preprocessing, clipping, and metric implementation are used for all learned models.

Performance over multiple operating points is summarized using Bjøntegaard delta rate (BD-rate). BD-rate measures the average relative bitrate difference between two rate–distortion curves over their common quality interval. Negative BD-rate indicates bitrate savings relative to the reference method at comparable reconstruction quality, whereas positive values indicate a bitrate increase. Following the conventional Bjøntegaard procedure [15], a cubic polynomial is fitted to the logarithm of bitrate as a function of the quality metric, and the difference between the fitted curves is integrated over their common quality interval before converting the result back to a relative bitrate percentage. The same procedure and overlapping quality interval are used for all methods, and the reference method is explicitly specified in each comparison. For BD-rate evaluation based on MS-SSIM, the similarity value is first transformed to the decibel domain:

(21) $$Q_{\mathrm{MS}}=-10\log_{10}\left(1-\mathrm{MS}\text{-}\mathrm{SSIM}\right).$$

BD-rate is then computed on the bpp–$Q_{\mathrm{MS}}$ curve over the common quality interval.

For the complexity comparison, the counting boundary is restricted to neural modules executed during decoding. It includes the hyper-synthesis network, the entropy-parameter network or spatial context network when present, and the synthesis transform. Encoder-side analysis and hyper-analysis transforms are excluded because they are not executed during decoding. Arithmetic-coder operations are also excluded from GMACs but remain included in the end-to-end latency measurement. The same decoding-side boundary is applied to every learned method.

For each counted convolutional layer *l*, including its bias term,

(22) $$P_{l}=k_{l}^{2}C_{l}^{\mathrm{in}}C_{l}^{\mathrm{out}}+C_{l}^{\mathrm{out}},\;P_{\mathrm{decode}}=\sum_{l\in D}P_{l},$$

where D denotes the set of neural layers executed during decoding.

The nominal convolutional workload is reported as multiply–accumulate operations (MACs). For layer l∈D,

(23) $${\mathrm{MACs}}_{l}=H_{l}W_{l}k_{l}^{2}C_{l}^{\mathrm{in}}C_{l}^{\mathrm{out}},\;{\mathrm{MACs}}_{\mathrm{decode}}=\sum_{l\in D}{\mathrm{MACs}}_{l}.$$

For pixel shuffle blocks, $C_{l}^{\mathrm{out}}$ denotes the pre-shuffle convolution output width. One multiply–accumulate is counted as one operation, and the same convention is used for every learned model. Values are reported in giga MACs (GMACs). This convention is kept separate from wall-clock latency.

Activation sparsity is evaluated independently of nominal GMACs. For constrained layer *l*, the proportion of low-magnitude responses at threshold ε is

(24) $$\rho_{l}\left(\varepsilon \right)=\frac{\#\left(\left|a_{l}\right|<\varepsilon \right)}{H_{l}W_{l}C_{l}}.$$

To obtain a model-level statistic without assigning equal weight to feature maps of different sizes, the reported global sparsity is computed using element-weighted aggregation:

(25) $$\rho_{\mathrm{global}}\left(\varepsilon \right)=\frac{\sum_{l=1}^{L_{c}}\#\left(\left|a_{l}\right|<\varepsilon \right)}{\sum_{l=1}^{L_{c}}H_{l}W_{l}C_{l}}.$$

In the reported sparsity analysis, the selected threshold is $\varepsilon^{⋆}=10^{-3}$. Both $\rho_{l}$ and $\rho_{\mathrm{global}}$ characterize feature-response statistics only; neither is interpreted as a reduction in the arithmetic operations executed by a conventional dense-convolution kernel. The threshold ε is used only for post-training sparsity statistics and does not alter optimization or inference.

#### 2.5.5. Decoding-Latency Measurement Protocol

All learned compression models are benchmarked with batch size 1 under the same hardware and software environment. Images are decoded individually at their native evaluation resolution. Before recording latency, 20 warm-up runs are performed to stabilize GPU execution and memory allocation. Each image is subsequently decoded 50 times.

End-to-end latency is measured with a CPU high-resolution wall-clock timer around the complete decoding function. The timing interval begins from in-memory entropy-coded bitstreams and ends after the reconstructed image has been produced. It includes hyperlatent entropy decoding, hyper-synthesis inference, primary latent entropy decoding, and synthesis transform inference. For autoregressive models, all position-dependent context-network evaluations required by sequential entropy decoding are included. Disk access, image loading, and output-file writing are excluded. GPU synchronization is performed immediately before starting the timed decoding call and immediately before reading the final timestamp so that asynchronous CUDA execution is included in the reported wall-clock time. To keep CPU-side entropy-decoding conditions identical across methods, entropy coding is executed with one CPU thread for all benchmarks; GPU inference uses the default CUDA stream.

For image *i*, the decoding time is first averaged over R=50 repeated runs:

(26) $${\bar{T}}_{i}=\frac{1}{R}\sum_{r=1}^{R}T_{i,r},\;R=50,$$

where $T_{i,r}$ denotes the wall-clock decoding time of image *i* in repetition *r*. For a test set containing *M* images, the reported dataset-level mean and standard deviation are then computed across the image-level means:

(27) $${\bar{T}}_{\mathrm{dataset}}=\frac{1}{M}\sum_{i=1}^{M}{\bar{T}}_{i},\;\sigma_{T}=\sqrt{\frac{1}{M-1}\sum_{i=1}^{M}{\left({\bar{T}}_{i}-{\bar{T}}_{\mathrm{dataset}}\right)}^{2}}.$$

Because image resolutions differ between datasets, resolution-normalized latency is computed on a per-image basis and then averaged:

(28) $$T_{\mathrm{MP}}=\frac{1}{M}\sum_{i=1}^{M}\frac{{\bar{T}}_{i}}{H_{i}W_{i}/10^{6}},$$

where $H_{i}$ and $W_{i}$ denote the height and width of image *i*, respectively, and $T_{\mathrm{MP}}$ is reported in milliseconds per megapixel.

#### 2.5.6. Ablation and Sensitivity Protocol

The factorial ablation baseline is not identical to the Minnen *joint* model used in the cross-method comparison. It retains the same analysis transform and latent dimensionality as the proposed framework, while NAE, LD, and AR are disabled. Spatial autoregressive conditioning and the standard capacity synthesis transform are restored, whereas all components unrelated to these three factors are kept unchanged. This construction isolates the effects of NAE, LD, and AR within a common experimental backbone.

The contributions of non-autoregressive entropy modeling (NAE), the lightweight decoder (LD), and activation regularization (AR) are evaluated using a complete $2^{3}$ factorial design. The eight evaluated configurations are Baseline, +NAE, +LD, +AR, +NAE + LD, +NAE + AR, +LD + AR, and the Full Model. This factorial design enables the individual and combined effects of the three components to be evaluated within a common experimental backbone.

Three sensitivity analyses are performed. First, the effect of activation regularization is evaluated by varying $\alpha_{\max}$ around the selected value $\alpha^{⋆}=1.0\times 10^{-4}$. Second, activation sparsity is recalculated using several values of ε around the selected threshold $\varepsilon^{⋆}=10^{-3}$. Third, decoder capacity is evaluated using three four-stage configurations that share the same spatial upsampling schedule and differ only in channel width: Decoder-S (160,128,96,64), Decoder-M (192,160,128,96), and Decoder-L (224,192,160,128). Decoder-M corresponds to the proposed configuration.

(29) $$\alpha_{\max}\in \left\{0,\;0.5\alpha^{⋆},\;\alpha^{⋆},\;2\alpha^{⋆}\right\},$$

(30) $$\varepsilon \in \{0.5\varepsilon^{⋆},\;\varepsilon^{⋆},\;2\varepsilon^{⋆}\}.$$

All hyperparameter and architecture choices are determined using the training/validation data and are not selected according to Kodak or BSD100 test performance. The three sensitivity analyses are summarized jointly in Section 3 so that the effects of $\alpha_{\max}$, ε, and decoder capacity can be directly compared under a consistent evaluation setting. The $\alpha_{\max}=0$ entry serves as the internal reference of the activation regularization sensitivity sweep; comparisons across $\alpha_{\max}$ values are therefore interpreted relative to this reference within the sweep.

## 3. Results

### 3.1. Rate–Distortion Performance on Kodak

Table 3 reports the five Kodak operating points used for the rate–distortion evaluation. To reduce redundancy, PSNR and MS-SSIM are presented as two panels within the same table. These operating points provide the numerical basis for the complete PSNR–bpp and MS-SSIM–bpp curves, while the corresponding BD-rate summary is reported jointly with BSD100 in the subsequent BD-rate comparison.

Across the overlapping bitrate range, the proposed model achieves consistently higher reconstruction quality than the hyperprior baseline while remaining below the stronger recent context-based methods. At approximately 0.30 bpp, the proposed model achieves 32.94 dB PSNR and 0.9745 MS-SSIM, compared with 32.55 dB and 0.9702 for the hyperprior method. In the subsequent BD-rate comparison, the proposed model obtains Kodak BD-rate reductions of 7.9% for PSNR and 9.7% for MS-SSIM relative to the hyperprior method. These results show that the reduction in decoding complexity is accompanied by a moderate rate–distortion trade-off relative to the strongest context-based models.

### 3.2. Rate–Distortion Performance on BSD100

Table 4 presents the five BSD100 operating points using the same two-panel organization as Kodak. Reporting Kodak and BSD100 in separate tables preserves dataset-specific rate–distortion characteristics, while the combined BD-rate table provides a concise comparison of the overall performance trends across the two datasets.

The BSD100 results show a ranking broadly consistent with that observed on Kodak, although the absolute reconstruction quality is lower on BSD100. Across the overlapping bitrate range, the proposed rate–distortion curve remains above the hyperprior baseline. At the operating point near 0.30 bpp, the proposed model achieves 31.95 dB PSNR and 0.9693 MS-SSIM at 0.313 bpp. The subsequent BD-rate comparison shows corresponding reductions of 6.4% for PSNR and 7.7% for MS-SSIM relative to the hyperprior method, indicating that the observed rate–distortion trend is not specific to the Kodak image distribution.

Figure 4 visualizes the complete rate–distortion behavior on Kodak and BSD100 using the five operating points reported in Table 3 and Table 4. The four panels separately show PSNR–bpp and MS-SSIM–bpp curves for the two datasets, allowing the comparison to be assessed over the full evaluated bitrate range rather than at a single representative operating point.

### 3.3. BD-Rate Comparison

To avoid duplicating nearly identical dataset-specific tables, the Kodak and BSD100 BD-rate results are consolidated in Table 5. The hyperprior method is used as the common reference method in both datasets.

### 3.4. Complexity and Decoding Latency

Table 6 consolidates the representative operating-point comparisons for Kodak and BSD100. The Kodak panel reports rate–distortion performance together with decoding-side parameter count, nominal GMACs, and measured latency, whereas the BSD100 panel reports dataset-specific rate–distortion and latency results to assess whether the observed efficiency trends remain consistent across different image content.

On Kodak, the joint model provides stronger rate–distortion performance but requires 710.3±16.7 ms per image because of serial autoregressive context inference. The proposed model reduces this latency to 35.6±1.0 ms, corresponding to an approximately 95.0% reduction in decoding time. The proposed decoding path also uses 8.10 M parameters and 165.3 GMACs, substantially below the other evaluated learned models. Dual-Branch is faster in absolute runtime at 23.6±0.9 ms, whereas the proposed model has the lowest parameter count and nominal GMACs among the learned methods. On BSD100, the same qualitative ordering is maintained after normalization by image size, supporting the consistency of the latency comparison across the two test sets.

Figure 5 further summarizes the representative quality–efficiency trade-off on Kodak. The latency panel highlights the large separation between the serial autoregressive joint baseline and the non-autoregressive methods, while the nominal-GMAC panel shows the arithmetic cost position of the proposed decoder. The plots are intended to characterize the combined operating point rather than to claim dominance in every individual metric.

### 3.5. Factorial Ablation Study

Table 7 reports the complete $2^{3}$ factorial ablation covering all combinations of NAE, LD, and AR. Because the configuration names directly identify the active components, the table enables the individual and combined effects of the three components to be evaluated within a common experimental backbone without introducing redundant configuration information.

Replacing serial spatial context with NAE reduces decoding latency from 681.5±15.9 ms for the baseline to 43.0±1.1 ms when NAE is used alone. LD reduces the parameter count from 16.50 M to 8.45 M and nominal GMACs from 412.6 to 235.4 when used alone. AR increases global activation sparsity from 11.7% to 36.8%, while latency changes only from 681.5±15.9 ms to 680.8±16.1 ms, which is within the observed measurement variation. The combined configurations further show that NAE and LD provide complementary efficiency gains, whereas AR primarily changes activation statistics rather than dense arithmetic.

### 3.6. Sensitivity Analysis

To provide a unified view of the main design sensitivities, the effects of activation regularization strength, activation threshold, and decoder capacity are consolidated in Table 8.

Increasing $\alpha_{\max}$ progressively raises global activation sparsity from 12.0% without AR to 54.9% at $2\alpha^{⋆}$, while the measured dense GPU latency remains nearly unchanged within the observed variation. Varying ε changes only the reported sparsity statistic, confirming that the threshold is a post-training analysis parameter rather than an inference control mechanism. For decoder capacity, Decoder-M provides the selected operating point: moving from Decoder-S to Decoder-M recovers approximately 0.22 dB, whereas increasing capacity further to Decoder-L yields only about 0.13 dB additional PSNR while substantially increasing parameter count, nominal GMACs, and latency.

## 4. Discussion

The experimental results show that the three components target different aspects of decoding efficiency rather than a single interchangeable notion of complexity. This distinction supports reporting activation sparsity, nominal GMACs, and wall-clock latency as separate efficiency indicators.

Across Kodak and BSD100, the proposed method performs between the hyperprior baseline and the stronger recent context-based methods in rate–distortion performance. Removing spatial autoregressive conditioning reduces part of the probability modeling gain available to context-based models, but it also removes the repeated position-dependent neural inference that dominates the latency of the serial autoregressive baseline. The rate–distortion results are therefore interpreted together with the corresponding differences in parameter count, nominal GMACs, and measured decoding latency. The proposed method is not the fastest method in absolute runtime: Dual-Branch achieves lower measured latency in the unified benchmark. However, the proposed model has the lowest parameter count and nominal GMACs among the evaluated learned codecs, indicating that its main advantage lies in the overall balance among runtime, parameter count, and nominal computation rather than in a single best metric.

The factorial ablation further confirms that the three components affect different aspects of the decoding pipeline. NAE accounts for most of the latency reduction, LD primarily reduces model size and nominal arithmetic workload, and AR substantially increases $\rho_{\mathrm{global}}$ while producing little change in dense GPU latency. This separation is consistent with the role assigned to each component in the method formulation and avoids interpreting activation sparsity as an automatic reduction in dense arithmetic.

The proposed framework is related to, but distinct from, previous efforts on efficient learned image compression. Parallel entropy modeling has been investigated to alleviate the sequential dependency of conventional autoregressive context models [5], while efficient transform architectures and space–channel contextual coding have been explored to improve the trade-off between compression performance and computational efficiency [6]. More recent approaches further reduce reliance on serial spatial context modeling through parallel or channel-wise conditional entropy coding [9]. In contrast, the present work evaluates entropy model dependency, decoder complexity, and activation regularization within a single decoding pipeline. Accordingly, the contribution lies in coordinating non-autoregressive entropy modeling, decoder simplification, and activation regularization within a decoding-oriented design and in experimentally separating their dependency-level, architecture-level, and representation-level efficiency effects.

A remaining limitation is that activation sparsity alone does not guarantee arithmetic reduction under conventional dense execution. Demonstrating sparsity-induced acceleration would require sparse kernels, zero-skipping hardware, structured pruning, or another execution backend capable of exploiting sparse responses. In addition, the desktop GPU benchmark characterizes relative decoding efficiency under a controlled environment but does not establish deployment performance on mobile or embedded hardware. Evaluation of latency, peak memory, and energy efficiency on resource-constrained devices remains an important direction for future work.

## 5. Conclusions

This paper presents a non-autoregressive learned image compression framework designed to reduce decoding latency and decoder complexity. The model combines dependency-decoupled entropy estimation, a lightweight synthesis transform, and activation regularization. On Kodak, the proposed model achieves 32.94 dB PSNR and 0.9745 MS-SSIM at 0.303 bpp with 8.10 M parameters, 165.3 GMACs, and a decoding latency of 35.6±1.0 ms. Relative to the serial autoregressive joint model at a comparable operating point, this corresponds to an approximately 95.0% reduction in decoding latency, together with substantial reductions in parameter count and nominal GMACs. On BSD100, the proposed model likewise remains above the hyperprior baseline over the overlapping bitrate range and achieves BD-rate reductions of 6.4% and 7.7% in terms of PSNR and MS-SSIM, respectively. The factorial ablation further shows that NAE accounts for most of the latency reduction, LD primarily reduces model complexity, and AR increases activation sparsity without producing a corresponding dense GPU speedup. Overall, the results demonstrate a practical trade-off between rate–distortion performance and decoding efficiency.

## Figures and Tables

**Figure 1:**
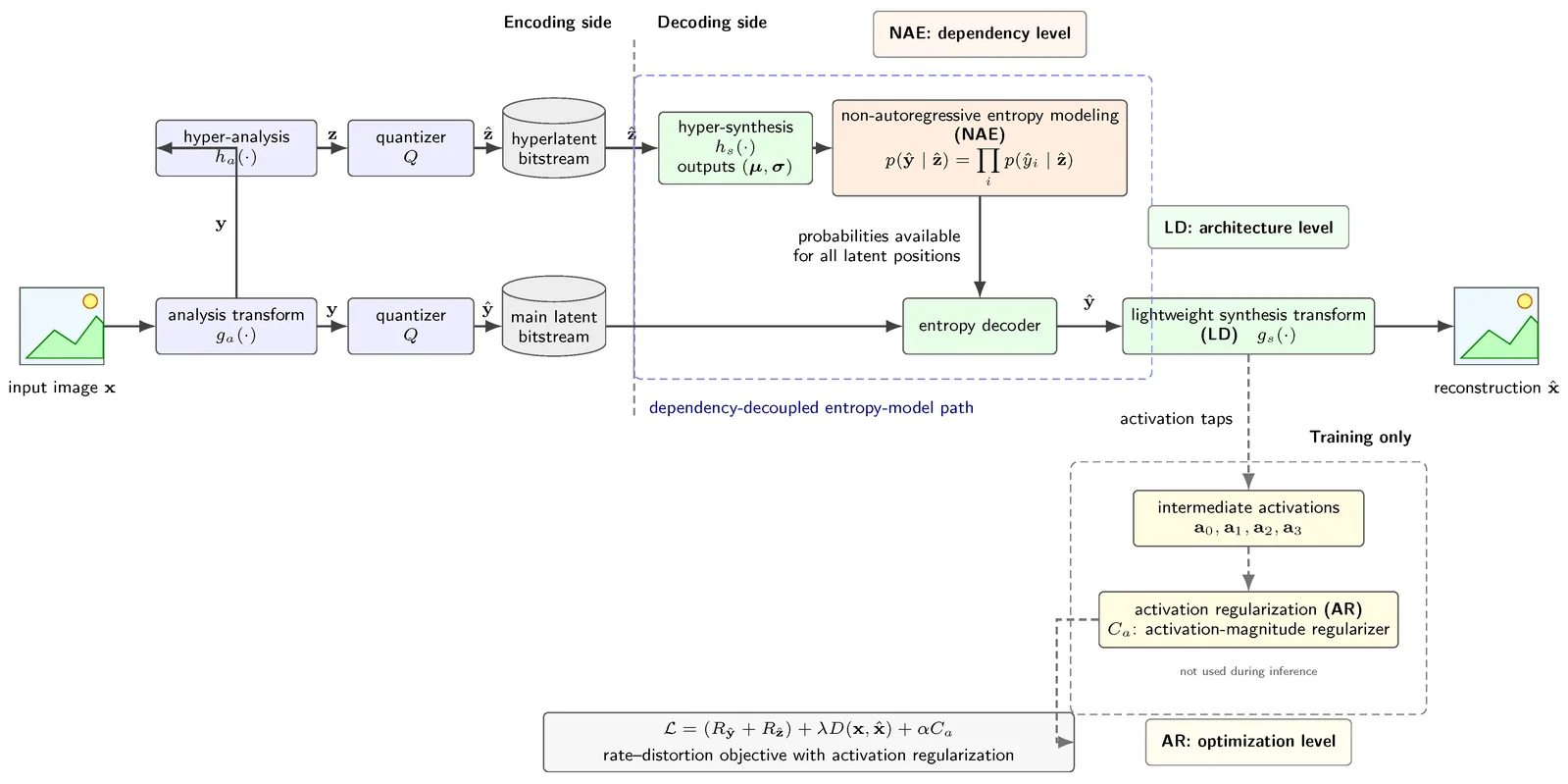
Overall architecture of the proposed learned image compression framework. The decoding pipeline combines non-autoregressive entropy modeling (NAE) to eliminate position-dependent neural context inference, a lightweight synthesis transform (LD) to reduce static reconstruction complexity, and activation regularization (AR) applied only during training. The dashed AR branch uses intermediate decoder activations to compute $C_{a}$ and does not introduce an additional inference path. Solid arrows indicate the main data flow, while dashed connections indicate auxiliary dependency or activation paths. Different colors are used to distinguish the encoding, decoding, NAE, and training-only AR components.

**Figure 2:**
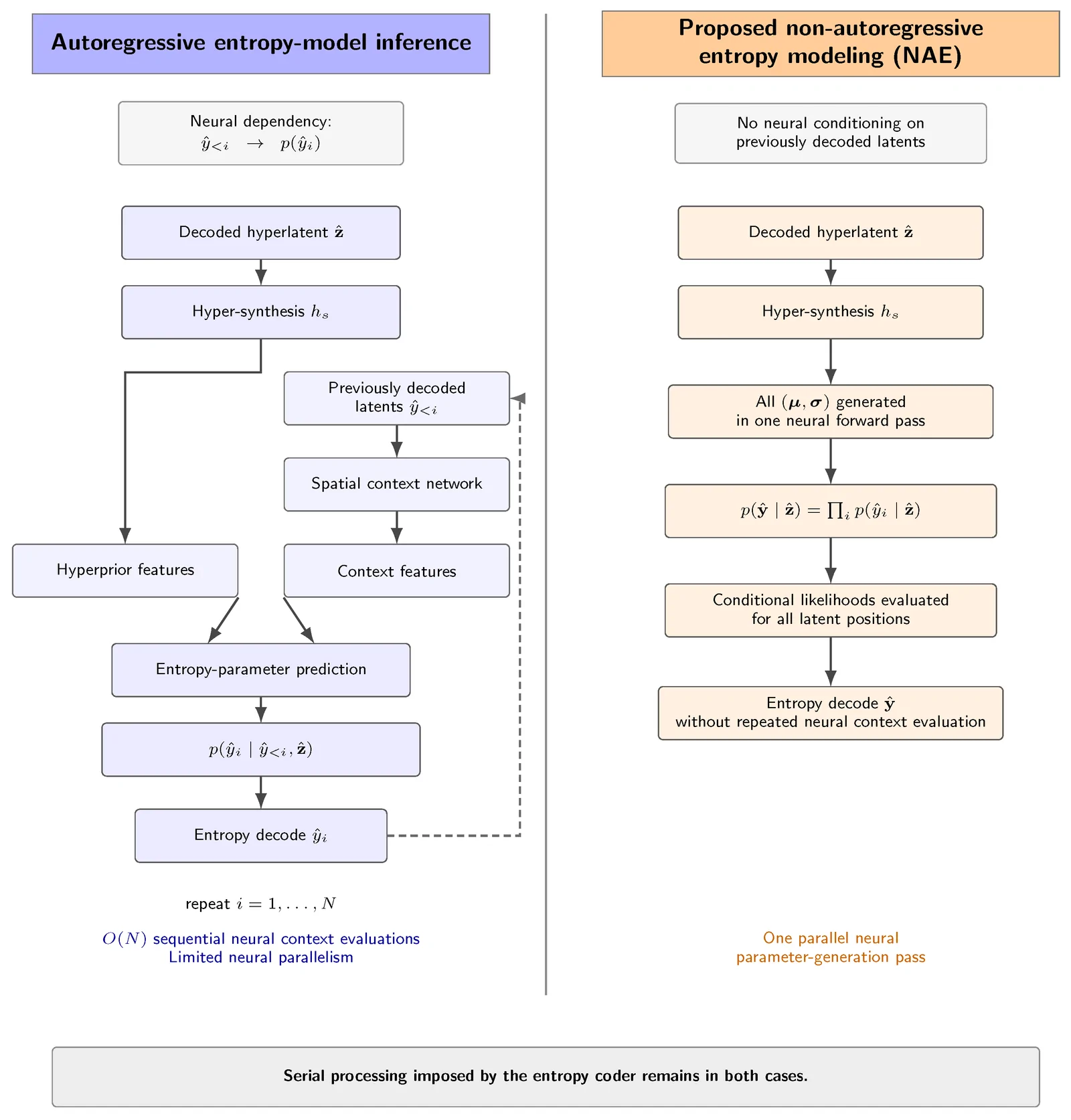
Comparison between autoregressive and proposed non-autoregressive entropy model inference. In the autoregressive model, hyperprior features and spatial-context features conditioned on previously decoded latents are jointly used to predict the probability distribution of each latent symbol, requiring repeated position-dependent neural context evaluations. In the proposed NAE, all distribution parameters are generated from the decoded hyperprior in one neural forward pass, and the conditional latent distribution is factorized without neural conditioning on previously decoded latents. This removes the neural autoregressive loop while serial processing imposed by the entropy coder itself remains unchanged. Purple and orange blocks distinguish the autoregressive and proposed non-autoregressive inference paths, respectively. Solid arrows indicate the forward information flow, while the dashed arrow denotes the repeated dependency on previously decoded latents in autoregressive inference.

**Figure 3:**
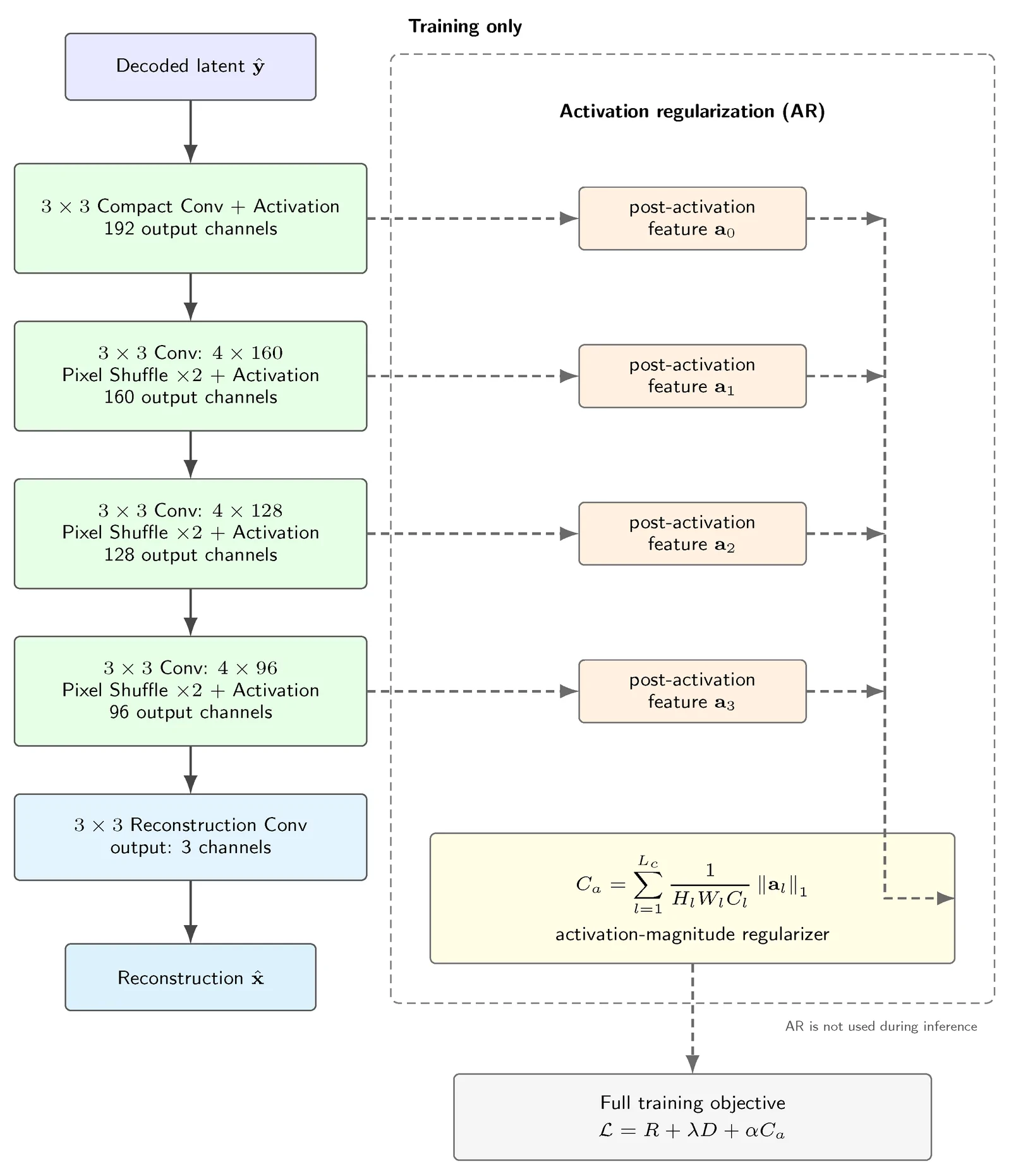
Architecture of the lightweight synthesis transform (LD) and the activation regularization branch used during training. The decoder uses a compact convolution followed by three progressive pixel shuffle upsampling stages and a final reconstruction convolution. Solid arrows indicate the main decoder forward path, while dashed connections indicate the post-activation feature tensors $a_{l}$ used to compute the activation-magnitude regularizer $C_{a}$. Different colors are used to distinguish the decoder stages, intermediate activation features, reconstruction output, and activation-regularization components. The AR branch is active only during training and introduces no additional operations during inference.

**Figure 4:**
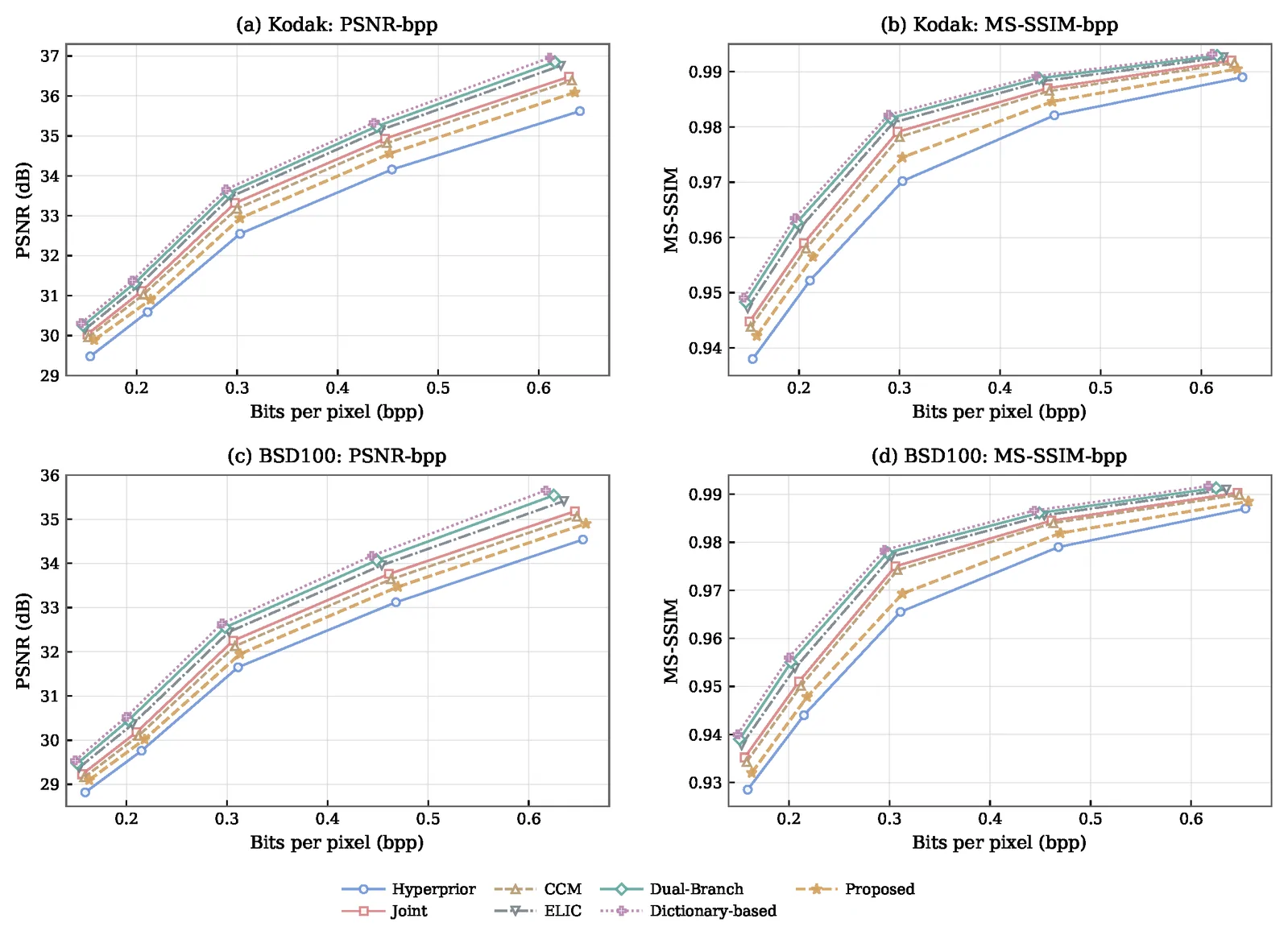
Rate–distortion performance of the compared learned image compression methods on Kodak and BSD100. Panels (**a**,**b**) show the PSNR–bpp and MS-SSIM–bpp curves on Kodak, respectively, while panels (**c**,**d**) show the corresponding results on BSD100. Each curve is constructed from five evaluated operating points. Across the overlapping bitrate range, the proposed model consistently improves upon the hyperprior baseline, while the stronger recent context-based methods retain higher pure rate–distortion performance.

**Figure 5:**
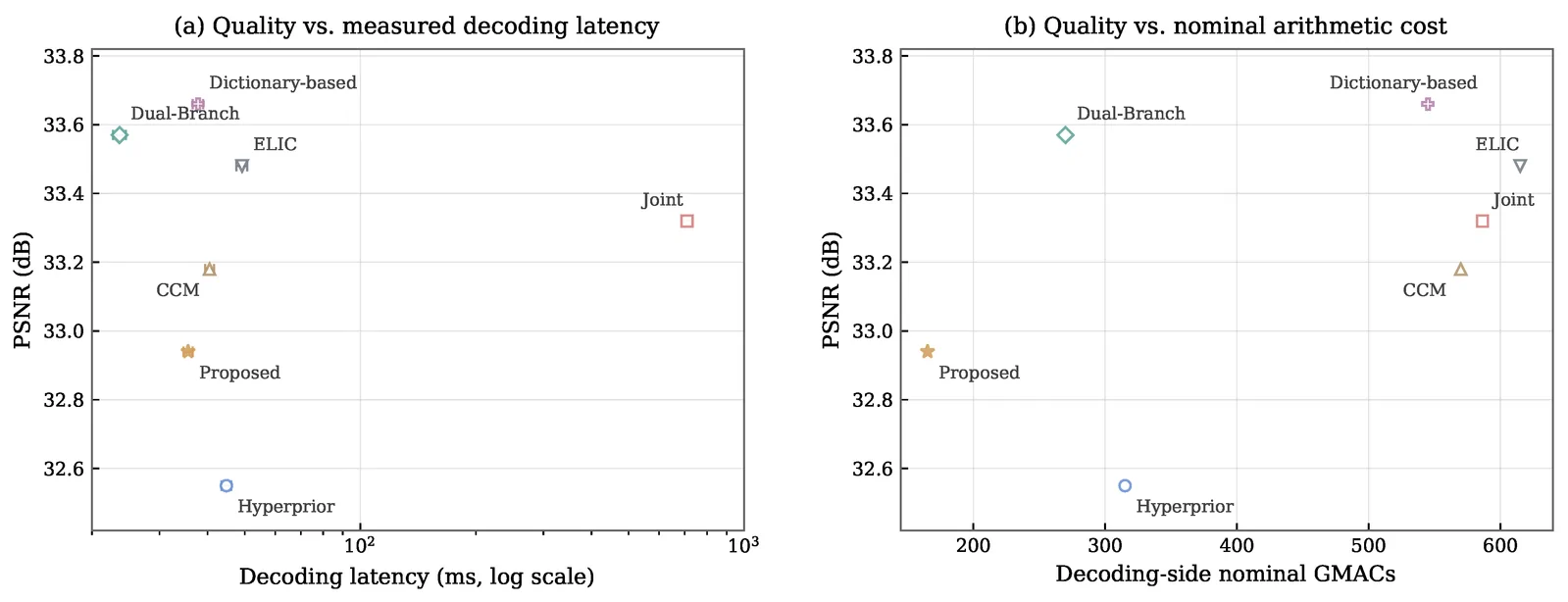
Quality–efficiency trade-off of the compared learned image compression methods at representative operating points near 0.30 bpp on Kodak. Panel (**a**) relates PSNR to measured decoding latency under the unified benchmark, with the latency axis shown on a logarithmic scale and horizontal error bars indicating the reported standard deviation. Panel (**b**) relates PSNR to decoding-side nominal GMACs. The proposed model does not achieve the highest PSNR or the lowest absolute latency; instead, it provides the lowest nominal GMAC count among the evaluated learned codecs while substantially reducing latency relative to the serial autoregressive joint baseline.

**Table 1:** Layer configuration of the proposed synthesis transform.

Stage	Operation	Kernel	Output Channels	Scale
$h_{0}$	Compact convolution	3×3	192	1×
$h_{1}$	Conv. + pixel shuffle	3×3	160	2×
$h_{2}$	Conv. + pixel shuffle	3×3	128	2×
$h_{3}$	Conv. + pixel shuffle	3×3	96	2×
Output	Reconstruction convolution	3×3	3	1×

**Table 2:** Experimental environment.

Category	Item	Configuration
Hardware	Processor	Intel Core i7-1360P, 12 cores/16 threads, up to 5.0 GHz
Hardware	GPU	NVIDIA GeForce RTX 3070, 8 GB GDDR6
Hardware	Memory	16 GB DDR5, 4800 MT/s
Software	Operating system	Ubuntu 20.04.6 LTS
Software	Framework	PyTorch 1.12.1, CUDA 11.3, cuDNN 8.4.1
Software	Language	Python 3.8.13
Software	Compression library	CompressAI 1.2.2 [10]

**Table 3:** Multi-rate performance on Kodak at five operating points. (**a**) PSNR–rate performance. Each entry is bpp/PSNR (dB). (**b**) MS-SSIM performance at the same operating points.

(**a**)					
**Method**	**Point 1**	**Point 2**	**Point 3**	**Point 4**	**Point 5**
Hyperprior	0.154/29.48	0.211/30.59	0.303/32.55	0.454/34.16	0.641/35.62
Joint	0.151/30.03	0.205/31.11	0.298/33.32	0.447/34.93	0.630/36.48
CCM	0.152/29.96	0.207/31.03	0.300/33.18	0.449/34.82	0.633/36.39
ELIC	0.149/30.16	0.201/31.23	0.294/33.48	0.443/35.15	0.622/36.76
Dual-Branch	0.147/30.25	0.198/31.31	0.291/33.57	0.439/35.24	0.616/36.85
Dictionary-based	0.145/30.31	0.196/31.38	0.289/33.66	0.436/35.33	0.611/36.96
Proposed	0.158/29.89	0.214/30.90	0.303/32.94	0.452/34.56	0.636/36.09
(**b**)					
**Method**	**Point 1**	**Point 2**	**Point 3**	**Point 4**	**Point 5**
Hyperprior	0.9380	0.9522	0.9702	0.9821	0.9890
Joint	0.9448	0.9590	0.9791	0.9870	0.9920
CCM	0.9438	0.9580	0.9782	0.9865	0.9916
ELIC	0.9472	0.9617	0.9808	0.9883	0.9926
Dual-Branch	0.9483	0.9627	0.9816	0.9888	0.9929
Dictionary-based	0.9491	0.9635	0.9822	0.9892	0.9932
Proposed	0.9422	0.9565	0.9745	0.9846	0.9905

**Table 4:** Multi-rate performance on BSD100 at five operating points. (**a**) PSNR–rate performance. Each entry is bpp/PSNR (dB). (**b**) MS-SSIM performance at the same operating points.

(**a**)					
**Method**	**Point 1**	**Point 2**	**Point 3**	**Point 4**	**Point 5**
Hyperprior	0.159/28.82	0.215/29.76	0.311/31.65	0.468/33.12	0.654/34.54
Joint	0.156/29.23	0.210/30.18	0.306/32.25	0.461/33.76	0.646/35.18
CCM	0.158/29.16	0.212/30.10	0.308/32.13	0.463/33.64	0.648/35.06
ELIC	0.153/29.38	0.206/30.36	0.302/32.45	0.454/33.96	0.635/35.41
Dual-Branch	0.151/29.47	0.202/30.45	0.298/32.55	0.449/34.07	0.625/35.54
Dictionary-based	0.149/29.54	0.200/30.53	0.295/32.64	0.444/34.17	0.617/35.65
Proposed	0.163/29.10	0.218/30.02	0.313/31.95	0.470/33.47	0.657/34.90
(**b**)					
**Method**	**Point 1**	**Point 2**	**Point 3**	**Point 4**	**Point 5**
Hyperprior	0.9285	0.9440	0.9655	0.9790	0.9870
Joint	0.9352	0.9510	0.9750	0.9845	0.9903
CCM	0.9343	0.9501	0.9742	0.9840	0.9899
ELIC	0.9378	0.9538	0.9770	0.9856	0.9910
Dual-Branch	0.9390	0.9550	0.9778	0.9861	0.9913
Dictionary-based	0.9400	0.9560	0.9784	0.9866	0.9917
Proposed	0.9320	0.9478	0.9693	0.9819	0.9885

**Table 5:** BD-rate performance on Kodak and BSD100 using hyperprior as the reference method.

Method	Kodak PSNR	Kodak MS-SSIM	BSD100 PSNR	BSD100 MS-SSIM
Hyperprior	0.0%	0.0%	0.0%	0.0%
Joint	−16.0%	−20.5%	−13.9%	−19.1%
CCM	−13.4%	−17.9%	−11.2%	−16.9%
ELIC	−20.0%	−26.2%	−19.0%	−24.4%
Dual-Branch	−22.4%	−28.9%	−22.0%	−27.3%
Dictionary-based	−24.3%	−30.9%	−24.3%	−29.4%
Proposed	−7.9%	−9.7%	−6.4%	−7.7%

**Table 6:** Representative rate–distortion, decoding-side complexity, and latency results near 0.30 bpp. (**a**) Kodak. (**b**) BSD100.

(**a**)							
**Method**	**bpp**	**PSNR**	**MS-SSIM**	**Time (ms)**	**ms/MP**	**Params (M)**	**GMACs**
Hyperprior	0.303	32.55	0.9702	44.8±1.3	113.9	11.85	315.2
Joint	0.298	33.32	0.9791	710.3±16.7	1806.4	22.45	586.4
CCM	0.300	33.18	0.9782	40.5±1.1	103.0	22.20	570.0
ELIC	0.294	33.48	0.9808	49.2±1.6	125.1	28.80	615.0
Dual-Branch	0.291	33.57	0.9816	23.6±0.9	60.0	15.20	270.0
Dictionary-based	0.289	33.66	0.9822	37.8±1.2	96.1	31.60	545.0
Proposed	0.303	32.94	0.9745	35.6±1.0	90.5	8.10	165.3
(**b**)							
**Method**	**bpp**		**PSNR**	**MS-SSIM**	**Time (ms)**		**ms/MP**
Hyperprior	0.311		31.65	0.9655	24.0±0.9		118.0
Joint	0.306		32.25	0.9750	372.0±11.5		1828.0
CCM	0.308		32.13	0.9742	21.7±0.8		106.7
ELIC	0.302		32.45	0.9770	26.5±1.0		130.3
Dual-Branch	0.298		32.55	0.9778	13.1±0.6		64.4
Dictionary-based	0.295		32.64	0.9784	20.5±0.8		100.8
Proposed	0.313		31.95	0.9693	19.1±0.7		93.9

**Table 7:** Factorial ablation results on Kodak near 0.30 bpp.

Model	bpp	PSNR	MS-SSIM	Time (ms)	Params (M)	GMACs	$\rho_{\mathrm{global}}$ (%)
Baseline	0.300	33.10	0.9753	681.5±15.9	16.50	412.6	11.7
+NAE	0.301	33.00	0.9748	43.0±1.1	15.80	385.2	12.1
+LD	0.301	33.03	0.9750	641.0±14.8	8.45	235.4	11.5
+AR	0.301	33.05	0.9751	680.8±16.1	16.50	412.6	36.8
+NAE + LD	0.302	32.98	0.9747	35.8±1.0	8.10	165.3	12.0
+NAE + AR	0.302	32.96	0.9746	43.2±1.2	15.80	385.2	38.7
+LD + AR	0.302	33.00	0.9748	640.5±14.7	8.45	235.4	35.9
Full Model	0.303	32.94	0.9745	35.6±1.0	8.10	165.3	40.2

**Table 8:** Sensitivity analyses on Kodak for activation regularization, sparsity threshold, and decoder capacity. (**a**) Sensitivity to activation regularization strength. (**b**) Sensitivity of global sparsity to ε. (**c**) Decoder capacity sensitivity.

(**a**)							
$\alpha_{\max}$		**bpp**	**PSNR**	**MS-SSIM**	$\rho_{\mathrm{global}}$ **(%)**	**Latency (ms)**	
0		0.301	33.00	0.9748	12.0	35.5±1.0	
$0.5\alpha^{⋆}$		0.302	32.98	0.9747	27.4	35.6±1.0	
$\alpha^{⋆}$		0.303	32.94	0.9745	40.2	35.6±1.0	
$2\alpha^{⋆}$		0.305	32.85	0.9740	54.9	35.8±1.1	
(**b**)							
ε				$\rho_{\mathrm{global}}$ **(%)**			
$0.5\varepsilon^{⋆}$				29.5			
$\varepsilon^{⋆}$				40.2			
$2\varepsilon^{⋆}$				52.1			
(**c**)							
**Decoder**	**Channels**	**Params (M)**	**GMACs**	**bpp**	**PSNR**	**MS-SSIM**	**Latency (ms)**
Decoder-S	(160,128,96,64)	5.45	118.7	0.306	32.72	0.9730	29.1
Decoder-M	(192,160,128,96)	8.10	165.3	0.303	32.94	0.9745	35.6
Decoder-L	(224,192,160,128)	12.25	239.8	0.301	33.07	0.9753	42.4

## Data Availability

The datasets used in this study are publicly available. Open Images is used for model training, while Kodak and BSD100 are used for evaluation as described and cited in Section 2.5.
